# Translational plausibility of honey-derived antimicrobial biomaterials against biofilm-associated infections

**DOI:** 10.3389/fmicb.2026.1971766

**Published:** 2026-10-26

**Authors:** António Machado, Juraj Majtan, José M. Álvarez-Suárez

**Affiliations:** 1 Laboratório de Biofilmes, Departamento de Biologia, Faculdade de Ciências e Tecnologia, Universidade dos Açores (UAc), Ponta Delgada, Portugal; 2 CIBIO, Centro de Investigação em Biodiversidade e Recursos Genéticos, InBIO Laboratório Associado, BIOPOLIS Programa em Genómica, Biodiversidade e Planeamento; Cátedra Unesco Terra no Mar: Biodiversidade e Sustentabilidade em Ilhas Atlânticas, Universidade dos Açores, Ponta Delgada, Portugal; 3 Laboratorio de Bacteriología, Instituto de Microbiología, Colegio de Ciencias Biológicas y Ambientales COCIBA, Universidad San Francisco de Quito USFQ, Quito, Ecuador; 4 Laboratory of Apidology and Apitherapy, Department of Microbial Genetics, Institute of Molecular Biology, Slovak Academy of Sciences, Bratislava, Slovakia; 5 Department of Microbiology, Faculty of Medicine, Slovak Medical University, Bratislava, Slovakia; 6 Laboratorio de Investigación en Ingeniería en Alimentos (LabInAli), Departamento de Ingeniería en Alimentos, Colegio de Ciencias e Ingenierías, Universidad San Francisco de Quito (USFQ), Quito, Ecuador

**Keywords:** antimicrobial resistance, biofilm, honey, topical application, wound

## Abstract

Chronic biofilm-associated infections drive antimicrobial resistance (AMR) via prolonged systemic antibiotic therapy that is often ineffective. Honeys from *Apis mellifera* and stingless bees (Meliponini) exhibit antimicrobial and anti-biofilm activity via complementary mechanisms: osmotic stress, low pH, hydrogen peroxide, methylglyoxal, bee defensin-1, and phenolic and organic compounds. This is most effective when concentrated at the application site. Yet honey therapeutics are often framed as systemic nutraceuticals despite biologically incoherent pharmacokinetics. We propose translational plausibility, an analytical framework built on four interdependent dimensions: spatial, temporal, dose, and compositional coherence that distinguishes microbiologically defensible interventions from speculative ones, applicable beyond honey to natural-product antimicrobials. Applied to honey, the framework identifies topical, localized application as the only translationally coherent clinical route. Reframed as ecological therapeutic biomaterials, honeys address interconnected AMR priorities: disrupting polymicrobial ESKAPE biofilms, preserving distant microbiotas, reducing systemic antibiotic use in wound care, potentiating antibiotics, and supporting stewardship. Planetary health benefits, pollinator conservation, meliponiculture, and equitable access emerge as corollaries, not primary justification.

## 1 Introduction

Antimicrobial resistance (AMR) is one of the defining public health crises of the 21st century. Global estimates attribute approximately 1.27 million deaths directly to bacterial AMR in 2019 and 4.95 million deaths associated with drug-resistant infections. Projections suggest that without coordinated action, the cumulative burden will escalate substantially through 2050 (Antimicrobial Resistance, 2022; World Health Organization, 2022). The World Health Organization’s Global Action Plan on Antimicrobial Resistance and the One Health Joint Plan of Action 2022–2026 have positioned AMR not as an isolated clinical phenomenon but as a systemic consequence of intensive antimicrobial use, ecological disruption, and inequitable access to effective therapies (World Health Organization, 2015; World Health Organization et al., 2022). Within this framing, AMR is simultaneously a medical, microbiological, ecological, and developmental crisis—one that cannot be resolved through the discovery of new systemic antibiotics alone, not least because the development pipeline for novel antibiotic classes has remained insufficient to tackle the growing clinical burden of AMR.

Although antimicrobial resistance arises from multiple interconnected factors, including antimicrobial misuse and overuse, transmission of resistant pathogens, and inadequate infection prevention and control, chronic biofilm-associated infections represent a particularly challenging clinical context. These infections frequently respond poorly to conventional antimicrobial therapy and may require prolonged or repeated treatment, potentially contributing to sustained antimicrobial selective pressure (Cangui-Panchi et al., 2022; Cangui-Panchi et al., 2023; Cangui-Panchi et al., 2026). Diabetic foot ulcers, venous leg ulcers, pressure injuries, infected burns, surgical site infections, and device-associated infections are paradigmatic examples: their management frequently relies on prolonged or repeated antibiotic courses, often with limited clinical benefit while exerting sustained selective pressure on patient and environmental microbiotas (Edwards-Jones, 2020; Senneville et al., 2024). Biofilms, structured microbial communities encased within a self-produced extracellular polymeric substance (EPS) matrix, can confer susceptibility reductions of up to a thousand-fold relative to planktonic counterparts. This occurs through mechanisms encompassing restricted antimicrobial penetration, altered metabolic states including the formation of persister cells refractory to bactericidal activity, and horizontal gene transfer events that propagate resistance determinants within the community (Stewart and Costerton, 2001). This creates a structural mismatch between the pharmacology of systemic antimicrobials and the biological realities of the infections they target—one that amplifies AMR without resolving infection. The ESKAPE pathogens—*Enterococcus faecium*, *Staphylococcus aureus* [including methicillin-resistant *S. aureus* (MRSA)], *Klebsiella pneumoniae*, *Acinetobacter baumannii*, *Pseudomonas aeruginosa*, and *Enterobacter* species—account for a major proportion of life-threatening multidrug-resistant (MDR) healthcare-associated infections. Their ability to form robust biofilms, tolerate oxidative stress, exploit quorum sensing (QS) for collective virulence, and transfer resistance determinants makes them particularly difficult to treat (Koo et al., 2017; Mulani et al., 2019).

It is important to note, however, that polybacterial infection does not necessarily equate to the presence of a true multi-species biofilm. *In vivo* visualization of chronic wound biofilms using PNA-FISH and confocal laser scanning microscopy has revealed that bacterial aggregates are predominantly mono-species in composition, with spatially segregated communities of *P. aeruginosa* and *S. aureus* co-existing within the same wound but forming distinct single-species microcolonies (Kirketerp-Moller et al., 2008; Fazli et al., 2009). This spatial partitioning has critical therapeutic implications: inter-species interactions inferred from co-isolation data may not manifest *in vivo* if the organisms occupy separate ecological niches within the wound bed (Kvich et al., 2020). The competitive fitness of individual bacterial species within a multi-species biofilm can also differ substantially from that observed under mono-species conditions, reflecting interactions that are complex, temporally dynamic, and modulated by environmental stressors (Ramstedt and Burmølle, 2022).

Against this backdrop, interest in natural antimicrobial biomaterials capable of delivering localized anti-biofilm activity—without the systemic selective pressure and microbiome disruption associated with broad-spectrum antibiotics—has grown considerably (Ng et al., 2014; Kalelkar et al., 2022). Honeys produced by *Apis mellifera* and by stingless bees of the tribe Meliponini occupy a distinctive position within this landscape. Both have been used therapeutically for millennia, with documented antimicrobial, anti-biofilm, and wound-healing activity reflecting the convergent biological logic of nectar-derived antimicrobial matrices (Vit et al., 2013; Scepankova et al., 2017). Medical-grade honey is among the best-established honey-derived therapeutic applications, with certain honey-based wound dressings marketed as regulated medical devices in specific jurisdictions (Jull et al., 2015). Yet alongside this translationally coherent application, the research literature has expanded substantially in a different direction. A proliferation of studies has reframed honeys as systemic nutraceuticals with antioxidant, anti-inflammatory, anticancer, neuroprotective, and cardiovascular indications, typically based on *in vitro* assays or animal models whose pharmacokinetic distance from human oral consumption is rarely examined (Erejuwa et al., 2012; Samarghandian et al., 2017; Cianciosi et al., 2018). This perspective argues that the dominant systemic narrative has distorted the translational future of honey-based therapeutics by directing disproportionate attention toward applications with insufficient translational plausibility while undervaluing the most microbiologically defensible ones. The future therapeutic relevance of honey lies not in increasingly speculative systemic nutraceutical claims but in translationally coherent local antimicrobial applications, and translational plausibility is the analytical instrument through which this argument can be made rigorous, generalizable, and falsifiable.

This Perspective does not aim to dismiss the biological activities of honey observed within *in vitro* or *in vivo* animal models. Rather, it argues that such activities should be interpreted through the lens of translational plausibility before they are converted into clinical or commercial narratives. Honey is used here as a model case to demonstrate how spatial; temporal, dose, and compositional coherence can distinguish therapeutically reachable applications from biologically interesting but clinically inaccessible bioactivities.

## 2 The translational paradox of systemic honey narratives

The scientific literature on honey and honey-derived products has grown substantially over the past three decades, and a meaningful proportion of this growth has occurred along an interpretive axis that frames honeys primarily as systemic nutraceuticals. Indexed publications present honeys as candidates for systemic antioxidant therapy, anti-inflammatory modulation, anticancer activity, neuroprotection, cardiovascular benefit, and metabolic regulation, typically extrapolating from *in vitro* assays or rodent models to project human oral consumption (Ahmed et al., 2018; Bolat et al., 2026). This trajectory reflects a broader tendency in natural product research in which laboratory bioactivities are progressively translated into clinical narratives through the language of dietary intervention (Cianciosi et al., 2018). The problem lies not in the experimental evidence itself, much of which is methodologically sound within its own scope, but in the inferential distance between the conditions under which a bioactivity is observed and the conditions under which it would need to operate to produce meaningful clinical benefit in humans.

Polyphenols, flavonoids, phenolic acids, and related compounds whose qualitative and quantitative profiles depend on floral and geographical origin, contribute substantially to honey’s antimicrobial, anti-biofilm, and anti-inflammatory bioactivity, acting independently and in synergy with other constituents such as hydrogen peroxide (Bucekova et al., 2026). The translational difficulty here is pharmacokinetic rather than mechanistic: *in vitro* and animal studies routinely use concentrations, or human-equivalent doses, that exceed plausible dietary intake (Reagan-Shaw et al., 2008; Nair and Jacob, 2016), while polyphenols themselves exhibit low intestinal absorption, extensive first-pass metabolism, and rapid plasma clearance, leaving circulating concentrations far below those required to reproduce the effects reported *in vitro* (Crozier et al., 2010; Cianciosi et al., 2018). These pharmacokinetic considerations raise important questions about whether the concentrations required to reproduce specific *in vitro* bioactivities can be achieved systemically through realistic dietary honey consumption, without necessarily excluding other potential systemic effects.

Compositional variability compounds the translational challenge in ways that systemic nutraceutical narratives seldom acknowledge. Honeys produced by *A. mellifera* vary substantially in phenolic content, antioxidant capacity, and antimicrobial profile depending on floral source, geography, season, processing, and storage (Becerril-Sanchez et al., 2021). Honeys produced by Meliponini exhibit additional variation, including higher water content, distinct sugar profiles, and fermentation-derived metabolites associated with their characteristic microbiota (Vit et al., 2025). Systemic nutraceutical narratives routinely generalize findings about specific honeys into claims about “honey” as a pharmacologically homogeneous category—a structural error that the compositional diversity of the matrix does not support, and that renders such claims both scientifically imprecise and clinically misleading. This systematic oversight is documented in the clinical honey literature, where most studies fail to report the botanical origin or biological functionality of the honeys employed (Majtan, 2024). We propose the term “translational plausibility” to denote the degree of biological and pharmacokinetic coherence between the conditions under which a bioactivity is observed and the conditions under which it would need to operate to produce a clinical effect. Findings can be experimentally robust and translationally implausible at the same time. The translational paradox of honey research lies in the continued promotion of systemic applications despite unresolved pharmacokinetic limitations, while localized antimicrobial therapy for chronic, biofilm-associated infections offers a more coherent pathway for clinical translation.

## 3 Translational plausibility as an analytical lens

Translational plausibility can be assessed through four interdependent dimensions: spatial, temporal, dose, and compositional coherence, each of which maps directly onto microbiological outcomes in the wound environment.

*Spatial coherence* refers to the alignment between the site where a bioactivity has been demonstrated and the site where the proposed clinical effect would need to occur. A bioactivity demonstrated against bacteria *in vitro* has clear spatial coherence with a topical wound application because the active compounds, target microorganisms, and site of effect occupy the same physical compartment—the wound surface and its associated biofilm. The same evidence invoked to support a systemic oral antimicrobial claim lacks spatial coherence because the compounds must survive gastrointestinal absorption, hepatic metabolism, and systemic distribution before reaching distant tissues, typically at concentrations orders of magnitude below those required for the observed effect.

*Temporal coherence* concerns the alignment between the persistence of active compounds at the site of action and the duration required for clinical effect. Many phenolic constituents of honeys exhibit plasma half-lives measured in hours, with metabolites rapidly cleared through renal and biliary pathways (Manach et al., 2004; Crozier et al., 2010). In contrast, topical wound applications maintain sustained local exposure for the duration of dressing contact, providing the prolonged antimicrobial activity required to impair mature biofilm structure and prevent recolonization.

*Dose coherence* evaluates whether concentrations achievable under realistic clinical conditions correspond to those required to produce the reported biological effect—the dimension at which the gap between *in vitro* evidence and systemic claims most frequently becomes quantitatively explicit (Reagan-Shaw et al., 2008; Nair and Jacob, 2016). In topical wound applications, dose coherence is ensured because the concentration at the site of action corresponds directly to that of the source material, unaffected by absorption or metabolism.

Finally, *compositional coherence* refers to the alignment between the material used to demonstrate bioactivity and the material proposed for clinical deployment. Findings obtained with Manuka honey are characterized by a specific compound, methylglyoxal (MGO), whose concentration (Green et al., 2022) cannot automatically be generalized to multifloral honeys from other regions, nor to honeys produced by entirely different bee lineages with distinct biochemical profiles. Topical wound applications permit the direct deployment of the characterized material, maintaining compositional integrity from experimental observation to clinical use.

Crucially, these four dimensions do not operate as independent checkboxes; they interact, and it is this interaction—not any single dimension in isolation—that determines translational plausibility. Spatial coherence without temporal coherence is insufficient: placing an active compound at the correct site for only a few minutes will not impair a mature biofilm regardless of concentration. Dose coherence without compositional coherence is similarly hollow: achieving a clinically relevant concentration of an uncharacterized or substituted material provides no assurance that the bioactivity originally demonstrated is the bioactivity being delivered. Topical wound applications are translationally coherent precisely because a single clinical act—applying a characterized honey-impregnated dressing to a wound—satisfies all four dimensions simultaneously: it establishes presence at the site of action, sustains that presence for the duration of dressing contact, delivers an undiluted and therefore clinically relevant concentration, and uses the same material whose bioactivity was characterized experimentally. Systemic applications of orally consumed honey, particularly those involving direct antimicrobial effects at distant tissue sites, face substantial challenges related to spatial, temporal, and dose coherence, as well as compositional variability. The translational plausibility of other proposed systemic effects should be evaluated according to their specific mechanisms, exposure requirements, and available clinical evidence.

Translational plausibility is best understood not as a replacement for but as a microbiologically specific instantiation of concepts already established in translational medicine. Biological plausibility asks whether a proposed mechanism is consistent with known physiology; translational plausibility asks the narrower and more operational question of whether the conditions under which that mechanism was demonstrated can be reproduced at the site, time, dose, and material identity required for clinical effect. Pharmacokinetic plausibility, long used to interrogate drug candidates before they enter costly clinical programs, maps closely onto the framework’s dose and temporal dimensions. Meanwhile, external validity, the standard by which preclinical findings are judged to generalize to real-world clinical populations and settings, maps onto its spatial and compositional dimensions. The framework also speaks directly to the well-documented “valley of death” separating preclinical promise from clinical efficacy: a substantial fraction of candidates that fail to cross this valley do so not because their target biology was wrong but because the preclinical evidence base supporting them was never translationally coherent to begin with. Translational plausibility differs from these adjacent concepts chiefly in its specificity to compositionally complex, route-of-administration-sensitive natural products, for which pharmacokinetic and external-validity frameworks designed largely around single-entity synthetic drugs do not straightforwardly apply.

This is not a problem unique to honey. Polyphenols, antioxidants, botanical extracts, phytochemicals, and nutraceuticals more broadly share the same structural vulnerability: laboratory bioactivities demonstrated under conditions of compositional purity, sustained exposure, and concentrations decoupled from achievable human pharmacokinetics are routinely extrapolated into systemic clinical narratives that the underlying pharmacology cannot support (Cianciosi et al., 2018). The recurrence of this pattern across unrelated natural product classes suggests that the failure is not idiosyncratic to any single compound or matrix but a generic consequence of how natural product research is incentivized: *in vitro* and rodent bioactivity is more easily generated, published, and cited than the pharmacokinetic and clinical work required to establish whether that bioactivity is reachable in practice. Translational plausibility makes this generic failure mode explicit and assessable, rather than treating each new natural product narrative as a novel scientific claim requiring independent re-litigation of the same translational gap.

Because the four dimensions are defined independently of honey’s specific chemistry, the framework generalizes directly to other antimicrobial biomaterials and natural-product-derived interventions for which translational claims are emerging—propolis, plant-derived essential oils, bacteriocins, and engineered biofilm-dispersal agents among them. In each case, the relevant question is not whether bioactivity exists but whether the site, duration, concentration, and material identity under which it was demonstrated can be reproduced at the point of clinical use. Applied prospectively, translational plausibility could function as a triage tool: a low-cost analytical step performed before committing resources to systemic clinical development of a natural product candidate, flagging at the outset which applications are worth pursuing and which are biologically precluded regardless of how compelling the underlying *in vitro* data appears. Used in this way, the framework’s value extends well beyond honey, offering a transferable diagnostic for a field in which compositional complexity has long been treated as a curiosity rather than as the central translational constraint it is. Figure 1 integrates the microbiological, mechanistic, and translational dimensions of honey-derived antimicrobial biomaterials for the treatment of biofilm-associated infections, linking antimicrobial mechanisms to the four dimensions of translational plausibility and to the downstream clinical and stewardship outcomes that follow from translationally coherent deployment.

**Figure 1:**
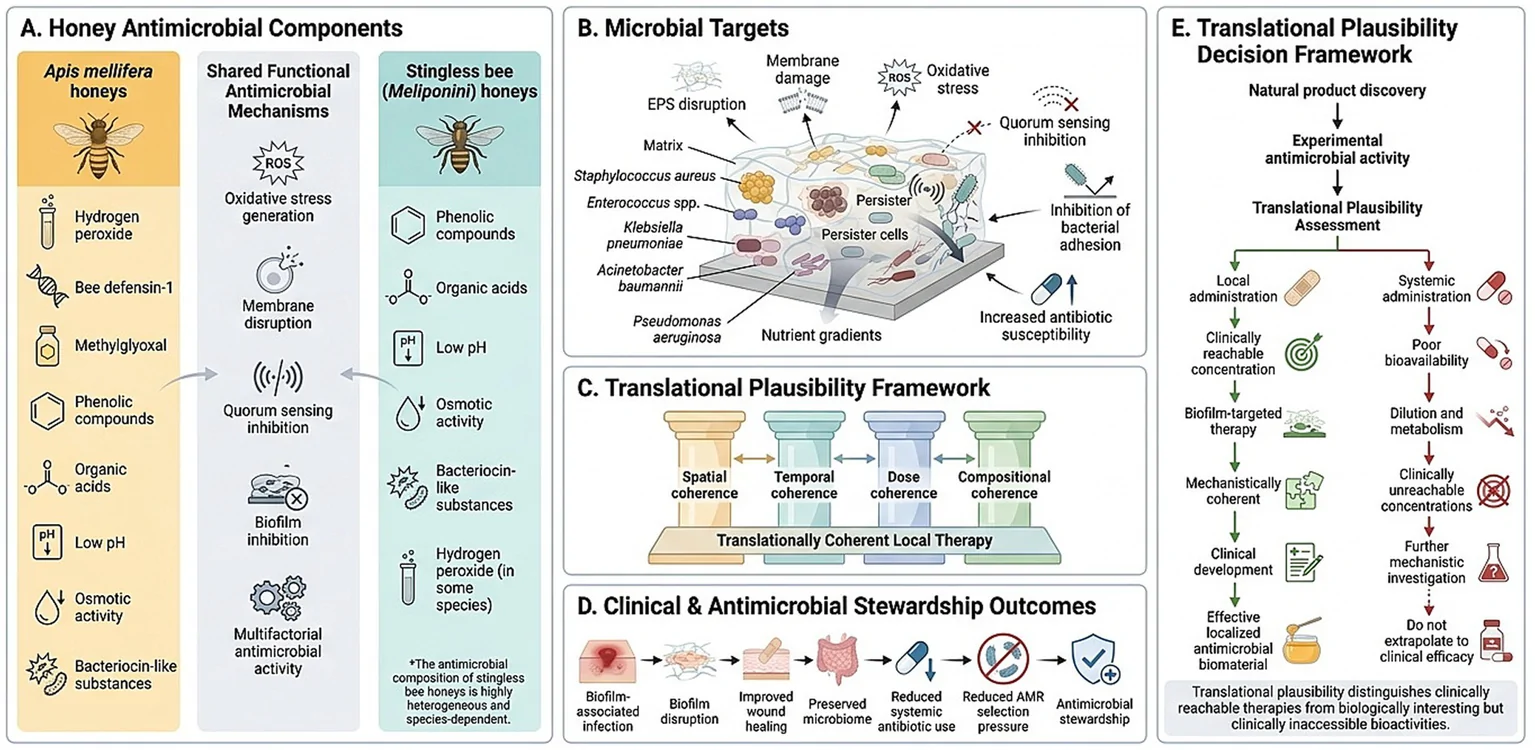
Translational plausibility framework for honey-derived antimicrobial biomaterials against biofilm-associated infections. The figure illustrates the proposed translational plausibility framework for evaluating honey-derived antimicrobial biomaterials and, more broadly, for assessing the clinical reachability of natural-product-derived therapeutics. **(A)** Honey antimicrobial components produced by *Apis mellifera* and stingless bees (*Meliponini*), highlighting the distinct and partially overlapping antimicrobial repertoires of both bee lineages. **(B)** Microbial targets within biofilm-associated infections, including ESKAPE pathogens and key biofilm features affected by honey-derived antimicrobial activity. **(C)** Translational plausibility framework: The four interdependent dimensions of spatial, temporal, dose, and compositional coherence that determine whether experimentally observed bioactivities can be translated into clinically reachable therapeutic effects. **(D)** Clinical and antimicrobial stewardship outcomes associated with translationally coherent localized therapies, including improved wound healing, biofilm disruption, reduced systemic antibiotic use, microbiome preservation, antimicrobial stewardship alignment, and reduced AMR selection pressure. **(E)** Translational plausibility as a decision framework: natural product discovery and experimental bioactivity are subjected to a translational plausibility assessment, creating a decision point between high-plausibility applications that warrant clinical development and low-plausibility applications requiring further mechanistic investigation. Honey topical therapy is presented as a worked example of the high-plausibility branch, whereas systemic nutraceutical claims illustrate the low-plausibility branch. Overall, the figure emphasizes that translational plausibility functions as a generalizable framework for distinguishing clinically reachable therapies from biologically interesting but clinically inaccessible bioactivities.

## 4 Localized therapeutics and translational realism

What follows is not presented as an inventory of honey’s bioactivity but as a worked illustration of how the four dimensions of translational plausibility operate jointly within a real, compositionally complex biomaterial. The same diagnostic exercise could equally be applied to propolis, essential oils, bacteriocins, or any other antimicrobial biomaterial whose translational claims rest on laboratory bioactivity rather than demonstrated coherence between site, duration, dose, and material identity; honey is the case study through which the framework’s mechanics are made concrete, not the boundary of its relevance.

### 4.1 Why local convergence of mechanisms matters

Honey’s antimicrobial activity does not depend on a single compound but on a constellation of mechanistically distinct agents—osmotic and oxidative stress, low pH, methylglyoxal, bee defensin-1, phenolic compounds, organic acids, and bacteriocin-like substances—that act simultaneously rather than redundantly (Machado et al., 2025; Majtan et al., 2025). The translational significance of this multiplicity is not that it is mechanistically interesting but that each of these agents reaches its effective concentration only where the undiluted material is in direct, sustained contact with the target microbial community. This condition is satisfied by a topical wound dressing yet violated by oral consumption, in which every one of these compounds is independently subject to dilution, metabolism, and clearance before it can reach a distant target. Honeys produced by Meliponini extend this repertoire with additional fermentation-derived metabolites and bacteriocin-like compounds largely absent from *A. mellifera* honeys (Cabezas-Mera et al., 2023). But the translational logic is identical regardless of bee lineage: compositional coherence requires that the specific material characterized in the laboratory is the material delivered to the wound, not a generalized pharmacological category of “honey” that the compositional diversity of the matrix does not support.

This same logic extends to the biofilm-specific activity most relevant to chronic wounds. Disruption of the extracellular polymeric matrix, inhibition of quorum sensing, and elimination of persister cell populations (Bouzo et al., 2020; Lima et al., 2023; Onyango and Liang, 2024) are mechanistically distinct from planktonic killing, but they share the same translational dependency as the mechanisms above: each requires sustained local exposure at undiluted concentration. Temporal and dose coherence is achievable only through topical application, and unreachable by any plausible systemic route. Multi-target activity of this kind also carries a translationally relevant corollary for resistance: given that no single mechanism is individually rate-limiting, the probability of stable resistance emerging through any one pathway is reduced, though not eliminated. Laboratory observations of transient honey tolerance under controlled conditions (Bischofberger et al., 2021) indicate that this remains an empirical question rather than a settled one, and a precautionary argument for continued surveillance rather than a challenge to the underlying translational logic (Combarros-Fuertes et al., 2020). The point of this discussion is not the catalog of mechanisms itself but *what* the catalog demonstrates: that honey’s antimicrobial profile becomes clinically meaningful only when delivered in a manner that preserves the spatial, temporal, dose, and compositional conditions under which it was characterized—conditions that topical application satisfies and systemic consumption structurally cannot.

### 4.2 Honey-antibiotic synergy: a combinatorial antimicrobial strategy

A clinically important extension of honey’s antimicrobial profile is its capacity to act synergistically with conventional antibiotics. Emerging evidence indicates that honey may potentiate antibiotic activity through complementary mechanisms: sub-inhibitory concentrations have been shown to lower minimum inhibitory concentrations against MDR bacteria, enhance antibiotic penetration into biofilm matrices, and attenuate the quorum-sensing networks that underlie collective antibiotic tolerance (Pourahmad Jaktaji and Ghalamfarsa, 2021; Balazs et al., 2023). Viewed through the framework developed above, this synergy is itself a dose-coherence phenomenon: it is only because undiluted honey can be maintained in sustained local contact with the biofilm (the same spatial and temporal conditions that make honey monotherapy translationally coherent) that sub-inhibitory concentrations can be reliably achieved and exploited as an adjunct. Conceptually, this matters beyond the laboratory because it reframes honey not as a substitute for antibiotics but as a dose-sparing adjunct: if sub-inhibitory honey concentrations can restore or potentiate antibiotic efficacy against resistant pathogens, lower antibiotic doses may achieve equivalent therapeutic effect, directly reducing the selective pressure exerted on commensal microbiotas. This positions honey-antibiotic combinations as a candidate strategy for antimicrobial stewardship protocols targeting pan-drug-resistant ESKAPE pathogens, and as a further demonstration that translational coherence, not bioactivity alone, is what converts a laboratory observation into a clinically actionable one. Evidence of this is still confined largely to *in vitro* systems but is mechanistically and stewardship-relevant enough to warrant validation in polymicrobial wound models and clinical trials.

### 4.3 Clinical evidence and stewardship implications

The clinical contexts in which translational coherence has been most clearly established are precisely the chronic, biofilm-associated infections that disproportionately drive the AMR burden: venous leg ulcers, diabetic foot ulcers, pressure injuries, burns, and selected mucosal infections. Systematic reviews and meta-analyses have evaluated the clinical effectiveness of honey-based wound treatments, particularly regarding wound healing, while evidence for other outcomes, including bacterial burden and pain relief, varies across studies (Yildiz Karadeniz and Kaplan Serin, 2023; Tang et al., 2024; Schils et al., 2026). This clinical convergence is not incidental to the framework; it is what translational plausibility predicts. The evidence base remains heterogeneous across honey types, formulations, and outcome measures. Yet the alignment of mechanistic rationale, clinical evidence, and regulatory recognition around the single application that satisfies all four dimensions of coherence is itself a structural marker of translational validity—one entirely absent from systemic nutraceutical narratives, whose clinical evidence base has not converged in the same way precisely because the underlying claims were never spatially, temporally, dose, or compositionally coherent to begin with.

The stewardship implications of this evidence extend beyond the wound itself. Chronic wounds affect a substantial proportion of populations in high-income countries, and their management frequently involves antibiotic courses that are prescribed empirically and prolonged well beyond microbiological necessity, generating selective pressure without corresponding clinical benefit (Serena et al., 2022; Sen, 2023). Repeated systemic antibiotic exposure disrupts gastrointestinal, oral, and cutaneous microbiotas, creating conditions for opportunistic colonization and propagating resistance determinants across the broader resistome (Schwartz et al., 2020; Fishbein et al., 2023). Localized antimicrobial interventions that resolve wound infection without perturbing distant microbiotas therefore advance antimicrobial stewardship at an ecological scale that extends well beyond the antibiotics they directly displace—a benefit that follows directly from spatial coherence since an intervention confined to the wound surface by design cannot exert the systemic collateral pressure that drives this disruption. Honeys are not proposed as a substitute for systemic antibiotics where these are genuinely indicated but as a translationally coherent alternative in the many cases where infection biology is fundamentally local—precisely the cases in which systemic therapy is least justified and most costly in resistance terms.

## 5 Outlook: translational discipline in the AMR era

The argument developed here is not, at its core, an argument about honey. It is an argument that natural-product research has, for decades, optimized for the wrong variable. The field has become extraordinarily efficient at generating bioactivity, new compounds, new assays, new mechanisms, and new correlations between *in vitro* effect and disease relevance while remaining comparatively undisciplined about asking whether any given bioactivity is reachable under the conditions in which it would need to operate clinically. Honey’s systemic nutraceutical literature is a particularly clear illustration of this asymmetry, but it is not an exception within natural-product science; it is closer to the field’s default mode. The future challenge for this domain is therefore not the discovery of additional bioactivities. It is the discipline to ask, before a bioactivity becomes a clinical narrative, whether it is biologically reachable at all—and the willingness to treat a negative answer as a finding rather than as an inconvenience to be elided in the discussion section.

This has direct consequences for how the field should now proceed with honey, and for what its example should teach researchers working on adjacent natural products. Multicenter randomized controlled trials have used standardized, well-characterized formulations, documented MGO content, phenolic profiles, anti-biofilm activity, and H_2_O_2_ output to combat defined wound types with microbiologically validated endpoints (biofilm disruption, ESKAPE pathogen load, wound microbiome composition, and systemic antibiotic consumption averted). These trials still constitute the most significant gap between mechanistic plausibility and clinical evidence for the one application that translational plausibility identifies as coherent. The standardized characterization of stingless bee honeys and adaptive regulatory pathways capable of accommodating ecologically variable, compositionally complex materials (rather than forcing them into standards built for single-entity synthetic compounds) are necessary precisely because compositional coherence cannot be assumed; it must be engineered into both the trial design and the regulatory category (Álvarez-Suárez et al., 2025; Álvarez-Suárez and Majtan, 2026; Álvarez-Suárez and Vit, 2026). None of this is specific to bees. Any natural-product field confronting compositional variability and route-of-administration sensitivity faces the same design problem and will require the same discipline to solve it.

Beyond its microbiological rationale, this reframing carries a planetary health corollary. Meliponiculture may incentivize pollinator conservation, while locally produced honeys could offer sustainable and accessible alternatives to imported antimicrobial dressings in regions where conventional wound care is constrained by cost, infrastructure, or supply chains. In this regard, translationally coherent honey-derived biomaterials may align AMR stewardship with biodiversity conservation and equitable access to wound care under a One Health framework.

Several limitations temper this outlook: clinical evidence remains heterogeneous, mechanistic findings require validation in complex polymicrobial environments that replicate *in situ* biofilm architecture, and no regulatory pathway yet exists for the compositional variability that defines Meliponini honeys. None of these limitations touches the central claim advanced here, which is methodological rather than empirical: that translational plausibility, not bioactivity, should be the threshold that a natural product crosses before its findings are permitted to become a clinical or commercial narrative. Applied retrospectively, the framework highlights the translational advantages of localized antimicrobial applications of honey and their potential for further clinical development. Applied prospectively to honey and to the wider pipeline of polyphenols, phytochemicals, botanical extracts, and nutraceuticals advancing through the same literature, it offers something the field currently lacks: a test that can be failed. In the AMR era, the scarcest resource is no longer a new bioactive molecule; it is the discipline to ask, of every promising one, whether biology would let it travel from bench to bedside, and to publish the answer when it will not.

This Perspective does not aim to provide an exhaustive catalog of honey bioactivities but rather to use honey as a model case to illustrate how translational plausibility can discipline the development of natural-product-derived antimicrobials. The systemic effects of orally consumed honey should not be dismissed *a priori*; however, they require a level of pharmacokinetic, dose–response, and clinical evidence that is currently much weaker than that supporting local wound applications.

## Data Availability

The original contributions presented in the study are included in the article/supplementary material, further inquiries can be directed to the corresponding author.
